# Blocking of the Lymphatic Vessel in Lymphedema

**Published:** 2017-03-27

**Authors:** Hisako Hara, Makoto Mihara

**Affiliations:** Department of Lymphatic and Reconstructive Surgery, Saiseikai Kawaguchi General Hospital, Saitama, Japan

**Keywords:** blocking, lymphaticovenous anastomosis, lymphedema, indocyanine green lymphography, ICG

## Abstract

**Objective:** In this case report, we present a case wherein we observed a blocking of lymphatic vessels in indocyanine green lymphography and found a shrunken lymphatic vessel intraoperatively. **Methods:** We performed indocyanine green lymphography and lymphaticovenous anastomosis on a 77-year-old woman. She had previously undergone right mastectomy and axillary lymph node dissection accompanied by radiotherapy and chemotherapy for right breast cancer. She noticed swelling in the right upper limb 22 years after the surgery and consulted our hospital. Although she started wearing elastic sleeve, there was still stiffness in the right upper limb, and we decided to perform lymphaticovenous anastomosis 5 months after the first consultation. **Results:** In the preoperative indocyanine green lymphography, we observed a linear pattern in the medial side of the right forearm, which suddenly blocked in the middle of the forearm. At that point, we observed dilated lymphatic vessels that were suddenly shrunken at the proximal side intraoperatively. We performed lymphaticovenous anastomosis with the dilated part of this lymphatic vessel. We also performed 4 additional lymphaticovenous anastomoses. The operation time was 2 hours 10 minutes, and the amount of bleeding was minimal. The right upper limb of the patient got softer, and she was satisfied with the result 3 months after the operation. The average circumference change at the 5 points in the right upper limb was −1.26 cm (range, −2.3 to −0.3 cm). **Conclusions:** There was a possibility that the blocking of the lymphatic vessels was a cause of lymphedema in the upper extremity.

It is known that hypertrophy of adipose tissue, fibrosis in the skin and subcutaneous tissue, and degeneration of the lymphatic vessels are present in lymphedema.^1^^-^5 After lymphadenectomy or radiotherapy, lymphatic flow is dammed up and accumulation of lymphatic fluid in the distal lymphatic vessels occurs.^6^ In turn, this causes an increase in lymphatic inner pressure.^7^ However, it is still unknown how injury of the lymphatic vessels progresses.

In this case report, we present a case wherein we observed a disruption of lymphatic vessels in indocyanine green (ICG) lymphography and found a suddenly shrunken lymphatic vessel intraoperatively. This may be helpful when considering the mechanism of injury progression in the lymphatic vessels or when determining the operation site of lymphatic venous anastomosis (LVA).

## METHODS

**Figure 1:**
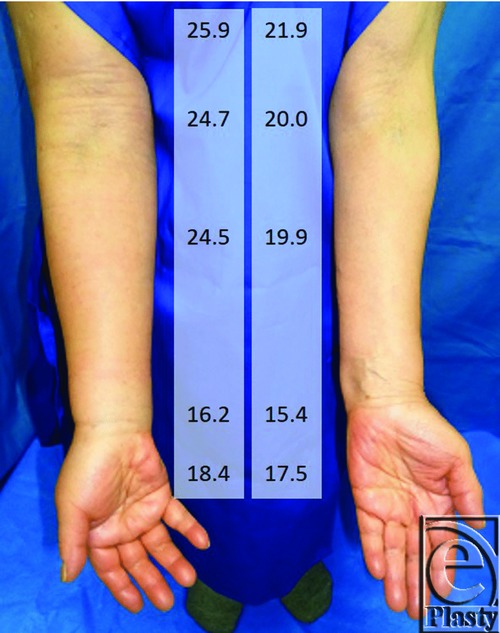
Preoperative photograph of the patient. Swelling is observed in the right upper limb. The numbers are circumference measurement at 10 cm proximal from the elbow, the elbow, 5 cm distal from the elbow, the wrist, and the hand (cm). Preoperative measurement was performed soon after the patient came to our hospital.

A 77-year-old woman underwent right mastectomy and axillary lymph node dissection accompanied by radiotherapy and chemotherapy for right breast cancer. She noticed swelling in the right upper limb 22 years after the surgery and consulted our hospital (Fig 1).

We performed ICG lymphography.^8^^,^^9^ To explain shortly, we injected 0.1 mL of ICG (0.5% Diagnogreen; Daiichi Pharmaceutical, Tokyo, Japan) subcutaneously into the second interdigital space of the right hand and the palmer side of the wrist and observed with an infrared camera system (Photodynamic Eye; Hamamatsu Photonics, Hamamatsu, Japan). We observed dermal backflow, which indicated the lymphatic stasis in the whole right upper limb and diagnosed our patient with lymphedema.^10^ Lymphoscintigraphy showed dermal backflow in the same area as ICG lymphotraphy. She started wearing elastic sleeve (20 mm Hg), and improvement in lymphedema symptoms was observed. As there was still stiffness in the right upper limb, which meant that there was still lymphatic stasis, we decided to perform LVA 5 months after the first consultation.

## RESULTS

**Figure 2:**
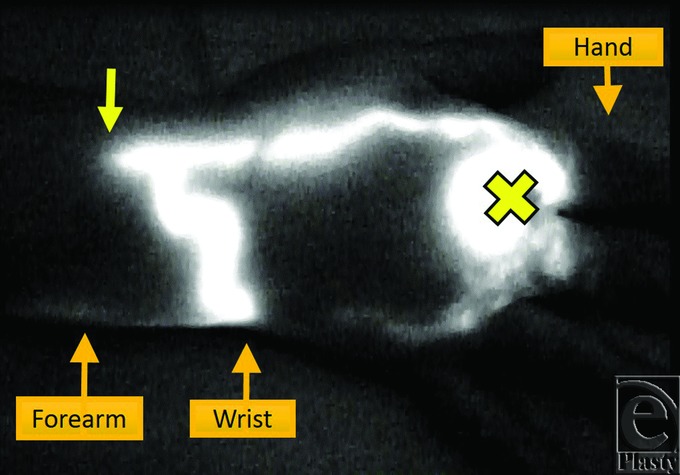
The finding of ICG lymphography. The right upper limb is located with the palm facing upward. ICG was injected at the palmar side of the right wrist (yellow cross). Clear linear pattern is observed that is suddenly disrupted at the middle of the forearm (yellow arrow). ICG indicates indocyanine green.

In the preoperative ICG lymphography to mark the location of the lymphatic vessels (preoperative day 1), we observed a linear pattern that indicated good lymphatic function in the medial side of the right forearm, which suddenly stopped in the middle of the forearm (Video). Although we massaged the arm and waited for more than 17 hours hoping ICG to move ahead, the disrupted line never advanced (Fig 2). The finding was similar on the next day, more than 17 hours after the injection. We decided to make a skin incision for LVA at the distal part of ICG disruption.

**Video:** Findings of preoperative ICG lymphography. Although a normal linear pattern could be seen in the radial side of the forearm at first, it suddenly stopped at the middle of the forearm. We observed progressive accumulation of ICG in the lymphatic vessel and the collateral lymphatics. ICG indicates indocyanine green.

**Figure 3:**
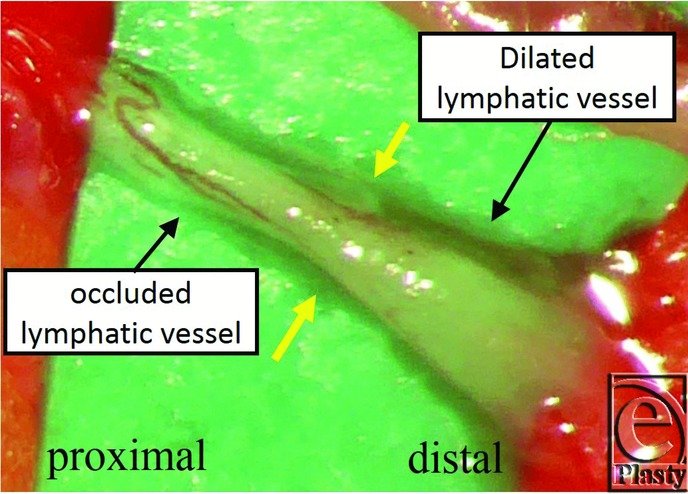
The intraoperative finding of lymphaticovenous anastomosis. Distal side of the lymphatic vessel is dilated and the proximal side is shrunk. The border between sides is indicated with yellow arrows.

At that point, we observed dilated lymphatic vessels that seemed to have a good lymphatic function intraoperatively. However, when we dissected the lymphatic vessel proximally, we found that it was suddenly shrunken (Fig 3). Although we waited for a while, considering the possibility of lymphatic spasm, it remained in the same condition. We concluded that this suddenly shrunken part of the lymphatic vessel was a cause of ICG disruption and lymphatic flow was blocked at this point; hence, we decided to perform LVA at the distal part of this lymphatic vessel. We also performed 4 additional LVAs using 2 surgical microscopes with 2 surgeons (H.H. and M.M.). The conditions of the other lymphatic vessels were as follows: normal type for 1 lymphatic vessel; ectasis type for 2; contraction type for 1; and sclerosis type for 1. The operation time was 2 hours 10 minutes, and the amount of bleeding was minimal.

**Figure 4:**
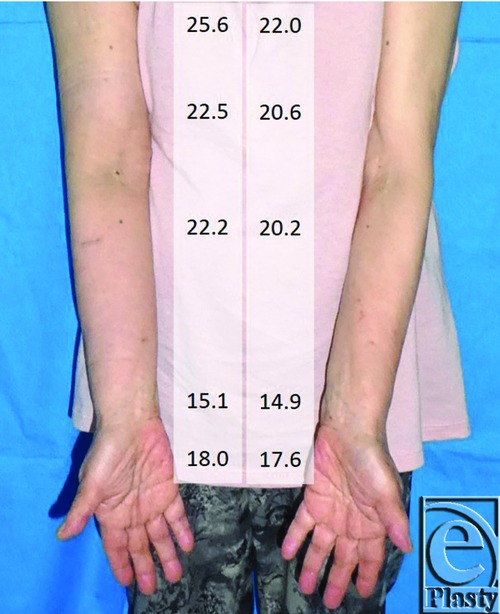
Postoperative photograph of the patient. Improvement in lymphedema symptoms was observed, and the forearm and upper limb got softer. The numbers are circumference measurement at 10 cm proximal from the elbow, the elbow, 5 cm distal from the elbow, the wrist, and the hand (cm). Postoperative measurement was performed soon after the patient came to our hospital.

There were no problems intra- and postoperatively. The patient started wearing compression sleeve 1 week after the operation, as she wore preoperatively. She did not undergo manual lymph drainage. The right upper limb of the patient got softer, and she was satisfied with the result 3 months after the operation (Fig 4). The average circumference change at the 5 points in the right upper limb was −1.26 cm (range, −2.3 to −0.3 cm), and the decrease was statistically significant with the Student *t* test (*P* = .042).

## DISCUSSION

In this case report, we presented a case of a suddenly shrunken lymphatic vessel at the site of disruption in ICG lymphography. To our knowledge, this is the first report showing this phenomenon.

Recently, degeneration of the lymphatic vessels in the extremities with lymphedema has been reported.^2^^,^^3^ Ogata et al^11^ reported transgenesis of smooth muscle cells. In the extremities with lymphedema, lymphatic vessels are dilated by abnormally increased inner pressure at the early phase. Subsequently, tunica media gets thick due to proliferation of smooth muscle cells and an increase of collagenous fiber, finally leading to complete occlusion.^3^ These changes do not occur in all of the lymphatic vessels simultaneously. The condition of the lymphatic vessels differs in different regions. Sometimes, different types of lymphatic vessels can be seen in 1 incision site. It is unclear whether lymphatic degeneration occurs gradually or suddenly. In addition, the reason for the different types of lymphatic vessels to appear in 1 region remains unknown. LVA is said to be the most effective when the lymphatic vessel of ectasis type is anastomosed.^8^ In addition, clarification of the mechanism of lymphatic degeneration or the location of the lymphatic vessels of ectasis type seems essential in the progression of LVA efficacy.

In the current case, we chose the incision site according to the findings of ICG lymphography, considering there may be some occlusive mechanism at the ICG disruption point. As expected, we could find a suddenly shrunken proximal part and the dilated distal part of the lymphatic vessel. This fact suggests that if we performed LVA at the distal side of the disruption in ICG lymphography, this may lead to good results of LVA.

There should be some hypothesis about the lymphatic failure mechanism in the present case. In usual cases, ICG flowing in the lymphatic vessels can move ahead with the support of manual lymph drainage even if the lymphatic vessel has pump failure. In the present case, ICG would not proceed despite our manual lymph drainage. The lymphatic vessel we found at that point intraoperatively, which is the next day of ICG lymphography, got sclerotic, not having an absolute lumen, and there was a dilation of the lymphatic vessel at the distal side. Therefore, we believe that there must have been some local occlusive mechanism in the lymphatic vessel. The mechanism lasted for about at least 12 hours, from the time of ICG lymphography to the operation, and it is not likely that it was due to a temporary lymphatic spasm.

This is only a case report and accumulation of the similar cases is needed. Also, we did not perform ICG lymphography intraoperatively in the present case, and it is hoped that intraoperative ICG lymphography findings will elucidate the lymphatic injury mechanism in the future.
